# Neglected Effects of Inoculum Preservation on the Start-Up of Psychrophilic Bioelectrochemical Systems and Shaping Bacterial Communities at Low Temperature

**DOI:** 10.3389/fmicb.2019.00935

**Published:** 2019-05-02

**Authors:** Sidan Lu, Binghan Xie, Bingfeng Liu, Baiyun Lu, Defeng Xing

**Affiliations:** ^1^State Key Laboratory of Urban Water Resource and Environment, School of Environment, Harbin Institute of Technology, Harbin, China; ^2^Department of Civil and Environmental Engineering, Louisiana State University, Baton Rouge, LA, United States

**Keywords:** bioelectrochemical system, low temperature, inoculum pretreatment, psychrophilic exoelectrogen, microbial fuel cell

## Abstract

Bioelectrochemical systems (BESs) are capable of simultaneous wastewater treatment and resource recovery at low temperatures. However, the direct enrichment of psychrophilic and electroactive biofilms in BESs at 4°C is difficult due to the lack of understanding in the physioecology of psychrophilic exoelectrogens. Here, we report the start-up and operation of microbial fuel cells (MFCs) at 4°C with pre-acclimated inocula at different temperatures (4°C, 10°C, 25°C, and −20°C) for 7 days and 14 days. MFCs with 7-day-pretreated inocula reached higher peak voltages than did those with 14-day-pretreated inocula. The highest power densities were obtained by MFCs with 25°C – 7-day-, 25°C – 14-day-, and 4°C – 7-day-pretreated inocula (650–700 mW/m^2^). In contrast, the control MFCs with untreated inocula were stable at 450 mW/m^2^. The power densities of MFCs with 7-day-pretreated inocula were higher than those obtained by MFCs with 14-day-pretreated inocula. The MFCs with 10°C – 7-day-pretreated inocula and the control MFCs showed higher chemical oxygen demand (COD) removal (90–91%) than other MFCs. Illumina HiSeq sequencing based on 16S rRNA gene amplicons indicated that bacterial communities of the anode biofilms were shaped by pretreated inocula at different temperatures. Compared with the control MFCs with untreated inocula, MFCs with temperature-pretreated inocula demonstrated higher microbial diversity, but did not do so with −20°C-pretreated inocula. Principal components analysis (PCA) revealed an obvious separation between the inocula pretreated at 4°C and those pretreated at 10°C, implying that bacterial community structures could be shaped by pretreated inocula at low temperatures. The pretreatment period also had a diverse impact on the abundance of exoelectrogens and non-exoelectrogens in MFCs with inocula pretreated at different temperatures. The majority of the predominant population was affiliated with *Geobacter* with a relative abundance of 17–70% at different pre-acclimated temperatures, suggesting that the exoelectrogenic *Geobacter* could be effectively enriched at 4°C even with inocula pretreated at different temperatures. This study provides a strategy that was previously neglected for fast enrichment of psychrophilic exoelectrogens in BESs at low temperatures.

## Introduction

Temperature is one of the main environmental factors that may potentially influence bacterial activities and wastewater treatment efficiency (Barria et al., 2013). Approximately 80% of natural climates on Earth, including the Arctic Circle and mountain areas, are permanently cold with temperatures below 5°C. Other areas are diurnal or seasonal in low temperatures (De Maayer et al., 2014). For example, the water temperatures in Arctic areas are as low as 5–10°C during winter (Lettinga et al., 2001). Besides natural environments, many discharged domestic and industrial wastewaters exist in low ambient temperatures (∼15°C), and contain complex pollutants that require effective cleaning (Petropoulos et al., 2019). With the exception of thermophilic anaerobic sludge digestion (55–60°C) (Dev et al., 2019), most biological wastewater treatments in the past decade have been tested in mesophilic conditions at 20–37°C because metabolisms are more active in mesophilic biological processes (Shrestha et al., 2018). However, to maintain a mesophilic condition, temperature-insulation or heat exchange materials, such as heat exchangers and plastic covers, are required for heating (Axaopoulos et al., 2001), and can potentially aggravate ongoing environmental problems. To date, the low efficiency of wastewater treatment is still a technical problem in colder regions because of the lack of information regarding anaerobic psychrophiles. Understanding psychrophilic microorganisms will be helpful for developing water treatment technology at low temperatures.

Bioelectrochemical systems (BESs) have the remarkable features of simultaneous wastewater treatment and resource recovery, low environmental impact, and mild operating temperatures (Arredondo et al., 2015). A typical reactor, such as microbial fuel cells (MFCs), can simultaneously remove organics and generate electricity from wastewater (Logan et al., 2006). In MFCs, exoelectrogens on the anode surface oxidize the organic or inorganic matters, and extracellularly transfer electrons that reach the cathode via an external circuit, which produces an electrical current. Currently, approximately 100 microbes from bacterial and archaeal domains are able to generate electricity by extracellularly transferring the electrons to an electrode acceptor (Costa et al., 2018). Based on MFCs, microbial electrolysis cells (MECs) were proposed for H_2_ production with a small applied voltage (Logan et al., 2008). Additionally, many other BESs were developed in membrane-combined configurations for wastewater treatment and energy recovery, including microbial desalination cells (Cao et al., 2009), microbial reverse-dialysis cells (Cusick et al., 2012), and microbial electrodialysis cells (Mehanna et al., 2010).

The possibility of applying BESs at low temperatures has been studied. Furthermore, direct and indirect acclimations have been developed to enrich psychrophilic biofilms at low temperatures (4–15°C). The first investigation reported the direct start-up and stable operation of MFCs and MECs at 4°C when a sequential inoculation method with a mixed-culture inoculum was employed (Lu et al., 2011). The indirect methods first needed to start BESs at a mesophilic condition (mostly 25°C and 30°C), and then the temperatures were decreased to 20°C, 10°C, or 5°C (Catal et al., 2011; Mei et al., 2017). Other methods used psychrophilic microbes as the inocula of BESs to treat wastewater at 15°C (Heidrich et al., 2018; Petropoulos et al., 2019). Although physiological characteristics, microbial growth rate, and microbial activity were reported to be negatively affected by a decreased temperature of wastewater (Zhou et al., 2018), psychrophilic bio-reactors have advantages that include lower operating costs, wide application, and advancement in enriching psychrophilic microbes compared with mesophilic conditions (Lu et al., 2012a; Xu et al., 2018). A microbial community of the electrode biofilms in BESs were reported to be noticeably shaped by temperature (Mei et al., 2017). Until now, many studies have reported the operation of psychrophilic BESs at 10–15°C, but few of the studies were operated at 4°C. Thus, the exploration of new methods that rely on simple operations to improve the feasibility of psychrophilic reactors is desperately needed. Additionally, a wider range of cold temperatures is also necessary for the application of bio-electrochemical technologies. In this study, the effects of inocula pretreated at different temperatures on the start-up and operation of MFCs at 4°C were estimated, and microbial community structures of the anode biofilms were analyzed using Illumina HiSeq sequencing of 16S rRNA gene amplicon.

## Materials and Methods

### Electrode Materials and MFC Configuration

The MFCs contained one chamber composed of polycarbonate cubes with an inner cylindrical configuration. The chamber was 3 cm in diameter and 4 cm in length with a volume of 25 mL. An anode and a cathode were placed inside the chamber, and no membrane was used to separate the space (Supplementary Figure S1). The anode was a graphite brush (3 cm in diameter × 3 cm in length) that was placed horizontally inside the left part of the MFC chamber. The anodes were successively washed in 1 M HCl (24 h), 1 M NaOH (24 h), and deionized water (24 h) to remove any possible pollutants. The anodes were later heated in a muffle furnace (Thermo Fisher Scientific) for 30 min at 450°C to attain higher N/C ratios in compositions (Feng et al., 2010). The air-cathode was a waterproof layer applied onto an oxygen reduction catalytic layer. For the waterproof layer configuration, a mixture of 44 mg carbon black (VulcanXC-72) and 20% polytetrafluoroethylene (PTFE) was applied on a circular piece of carbon cloth, and the area of the cathode was 7 cm^2^. The carbon cloth was then heated for 30 min in a muffle furnace (Thermo Fisher Scientific) at 370°C, and dried at room temperature. Next, 60% PTFE was smeared on waterproof layer of the cathode and heated at 370°C for 10 min. The procedure was repeated three times until the waterproof layer was finished. The catalytic layer was made after the waterproof layer was air-dried for 24 h. To make the catalytic layer, a mixture of 15 mg/cm^2^ Pt powder, 50 μL isopropanol, 100 μL Nafion, and 12.5 μL deionized water was applied on the other side. The graphite brush anode and the air-cathode were connected by a 1,000 Ω resistance in closed-circle. After the configuration, a data acquisition system (Keithley 2700) was connected to the anode and cathode to monitor the voltages.

### Inocula Pretreatment and MFC Operation

The MFCs were initially inoculated with a mixture composed in equal parts of the second sedimentary sludge collected from the Wenchang Wastewater Treatment Plant (Harbin, Heilongjiang, China) and the growth medium. Before the inoculation, the sludge was preserved in −20°C, 4°C, 10°C, and 25°C temperatures, respectively, in a refrigerator or thermal tank for cold acclimation. The preservation times for each temperature were 7 days and 14 days. All of the MFCs were operated in fed-batch mode in duplicate for each test. In total, eighteen reactors, including two control reactors, were started up and operated at 4°C. The control reactors were incubated with wastewater instantly collected from the wastewater treatment plant and without any preservation treatment. The growth medium fed to the MFCs contained 2 g CH_3_COONa, 11.55 g Na_2_HPO_4_, 2.77 g NaH_2_PO_4_ ⋅ 2 H_2_O, 0.31 g NH_4_Cl, and 0.13 g KCl in 1 L deionized (DI) water with some vitamins and minerals to fulfill the nutrient requirements of the microbes (Mei et al., 2017). The currents were measured based on I = U/R by monitoring the voltages across the 1,000 Ω resistor (R) using a data acquisition system that collected the voltage data at 10 min intervals. A voltmeter was used to ensure the validity of the data acquisition system. Once currents were produced, only the growth medium was fed to the reactors until stable currents were generated. Power density was measured by incrementally changing the external resistance ranging from 75 Ω to 3,000 Ω, and each resistance was maintained for 4 h to obtain three voltages for calculation.

### Chemical Analyses and Electrical Calculation

Soluble chemical oxygen demand (COD) was determined using test kits (TNT plus vial test, Hach Co.). The COD removal (%) was calculated as the ratio between the output mass of COD and the input mass in the MFCs. The electrical current was calculated according to U = IR. Power density was calculated based on W = UI/A (the electrode surface area, 7 cm^2^). Coulombic efficiency was calculated as the ratio between the experimental coulombs by integrating the current over time and the theoretical coulombs calculated based on COD changes. The equation for this calculation is *C* = *F* ⋅*b* ⋅*V* ⋅△_COD_, in which *C* = Coulombic efficiency, *F* = 96485 s A/mole, Faraday constant, *b* = 4, the exchanged number of electrons per mole O_2_, *V* = 25 mL, the volume of the anode liquid, and △COD = the differences of the COD changed in one cycle.

### DNA Extraction and High-Throughput Sequencing of 16S rRNA Gene Amplicons

The anode biofilm samples were collected after the graphite brushes had been run for over 2 months in satisfying operation. Carbon fiber pieces from the top, middle, and bottom positions of the graphite brushes were cut into small pieces by scissors and tweezers to collect the biofilm. The scissors and tweezers were washed with alcohol to prevent contamination. The brush pieces were transferred into a 1 mL centrifuge tube for future DNA extraction. PowerSoil DNA Isolation Kits (Mo Bio Laboratories, Inc., United States) were used for the DNA extraction experiment. After the extraction procedure, the DNA quality was detected via NanoDrop 8000 (Thermo Fisher Scientific, United States). Polymerase chain reaction (PCR) reactions were conducted using the bacterial primers 8F (5′-AGAGTTTGATCCTGGCTCAG-3′) and 533R (5′-TTACCGCGGCTGCTGGCAC-3′) to amplify the V1–V3 region of the 16S rRNA genes. The qualified samples were sequenced by Illumina HiSeq 2500 platform.

Raw reads after high-throughput sequencing were processed using the open-source software package Quantitative Insights into Microbial Ecology (QIIME)^1^ to remove the unqualified alignments. A 97% similarity was set as the confidence threshold to determine operational taxonomic units (OTUs). The Silva database^2^ was used to identify the representative sequence of each OTU. Diversity indices such as ACE, Chao1, and Shannon indexes were calculated by MOTHUR software. Rarefaction curves and principal components analysis (PCA) were conducted to visualize differences in the community structures of the anode biofilms in MFCs.

## Results

### Electricity Generation by MFCs With Different Pretreated-Inocula

**FIGURE 1:**
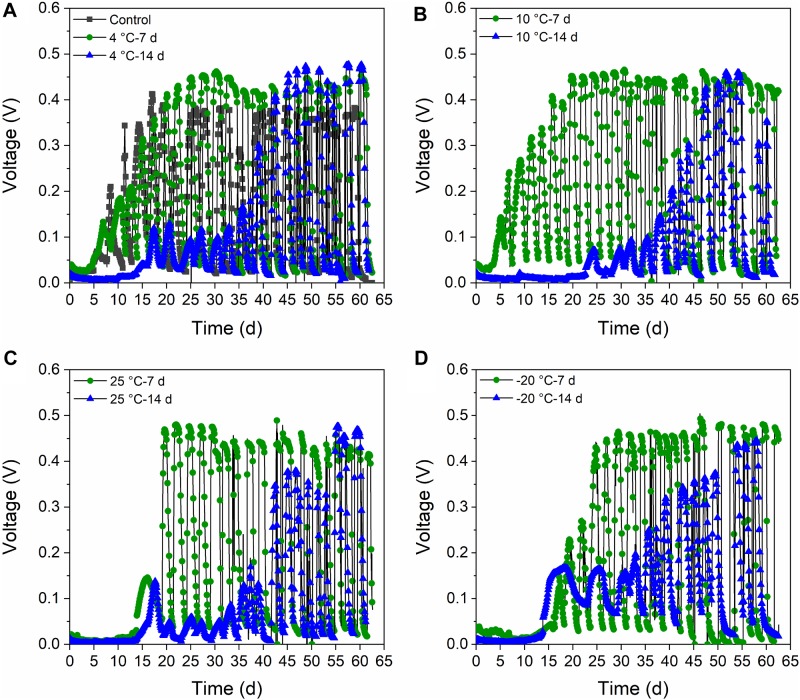
Voltage generation (external resistance of 1,000 Ω) of MFCs at 4°C with the inocula pretreated at different temperatures for 7 days and 14 days. **(A)** Control and active sludge pretreated at 4°C for 7 days and 14 days (4°C – 7 days and 4°C – 14 days). **(B)** Active sludge pretreated at 10°C for 7 days and 14 days (10°C – 7 days and 10°C – 14 days). **(C)** Active sludge pretreated at 25°C for 7 days and 14 days (25°C – 7 days and 25°C – 14 days). **(D)** Active sludge pretreated at –20°C for 7 days and 14 days (–20°C – 7 days and –20°C – 14 days).

The MFCs with 7-day-pretreated inocula attained a stable voltage production of ∼0.48 V after operation for 20 days. The voltages of the 10°C – 7-day and 4°C – 7-day MFCs increased faster than did the 25°C – 7-day and −20°C – 7-day MFCs (Figure 1). However, the voltage of the control reactors without pretreatment was very slow to increase, and only reached 0.4 V voltage at the stable stage. The voltages of the 14-day-treated reactors finally attained stability after 45 days of operation (0.48 V) (Figure 1). The −20°C – 14-day and 25°C – 14-day reactors reached higher voltages at the first several cycles. The 10°C – 14-day was the slowest to obtain stable peak voltages, and therefore differed from the 7-day-treated samples. However, the control reactors reached stable peak voltages earlier than the reactors with 14-day-pretreated inocula.

**FIGURE 2:**
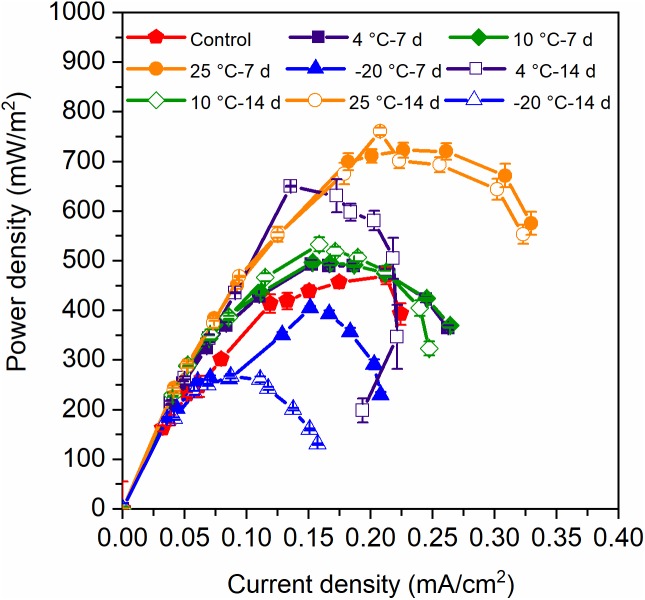
Power density of MFCs which were started up at 4°C with the inocula pretreated at different temperatures for 7 days and 14 days. Error bars represent standard deviation based on measurements from duplicate reactors in three batch cycles.

The voltage production of the MFCs with 7-day- and 14-day-pretreated inocula exhibited distinct acclimation, though the control reactors were the slowest and achieved the lowest peak voltage (Figure 1). It can be concluded that both the 7-day- and 14-day-pretreatments in the psychrophilic temperatures had positive impacts on the start-up process, and the MFCs with 7-day-pretreated inocula reached higher peak voltages than did those with 14-day-pretreated inocula. The highest power densities were confined to the MFCs with 25°C – 7-day- and 25°C – 14-day-pretreated inocula (700 mW/m^2^), while the 4°C – 7-day sample was lower than the 25°C temperatures (650 mW/m^2^) (Figure 2). In contrast, MFCs with −20°C – 7-day- and −20°C – 14-day-pretreated inocula obtained the lowest power densities of 200∼300 mW/m^2^ when the control MFCs were stable at 450 mW/m^2^, indicating that 7-day-pretreated inocula at temperatures that were higher than −20°C were helpful for extracellular electron transfer (EET) in MFCs. In total, the power densities of MFCs with 7-day-pretreated inocula were higher than those with 14-day-pretreated inocula, and the decrease of temperature indicated negative influence on the capacity of EET.

### Effect of Pretreated-Inocula on Organics Removal of MFCs

**FIGURE 3:**
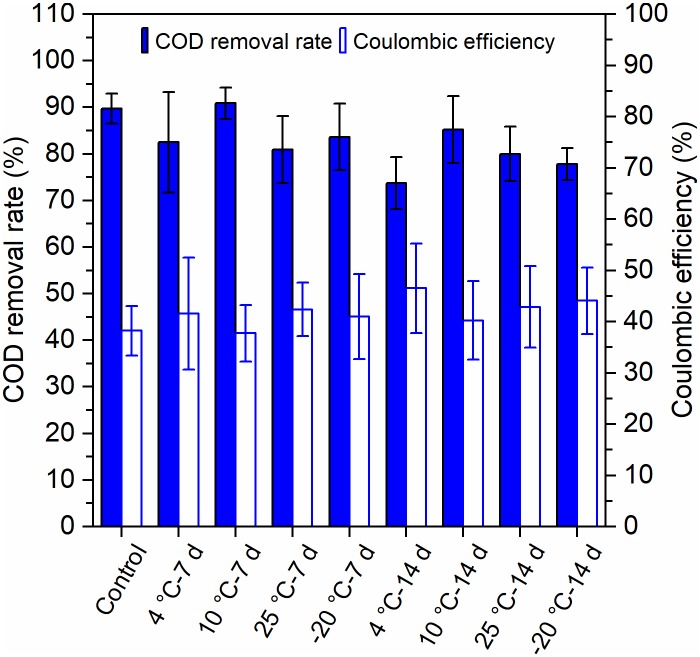
Removal of chemical oxygen demand (COD) and the coulombic efficiency (CE) of MFCs that were started up at 4°C with the inocula pretreated at different temperatures for 7 days and 14 days. Error bars represent standard deviation based on measurements from duplicate reactors in three batch cycles.

Both the pretreatments of 7 days and 14 days at temperatures of 4°C, 10°C, 25°C, and −20°C attained high COD removal rates (73–91%) (Figure 3). The COD removal of MFCs with 10°C – 7-day-pretreated inocula and the control reactors (90–91%) was higher than that of other MFCs, and the MFCs with 4°C – 14-day-pretreated inocula had the lowest COD removal of 73%. The coulombic efficiencies (CEs) of the MFCs fluctuated between 41 and 51%. The 4°C – 14-day-pretreated inocula exhibited the highest CEs, while the 10°C – 7-days and the control reactors (41–42%) exhibited the lowest (Figure 3). The MFCs with 7-day-pretreated inocula had higher COD removal, but lower CEs, than did the 14 days.

### Microbial Diversity of the Anode Biofilms in MFCs With Pretreated-Inocula

**FIGURE 4:**
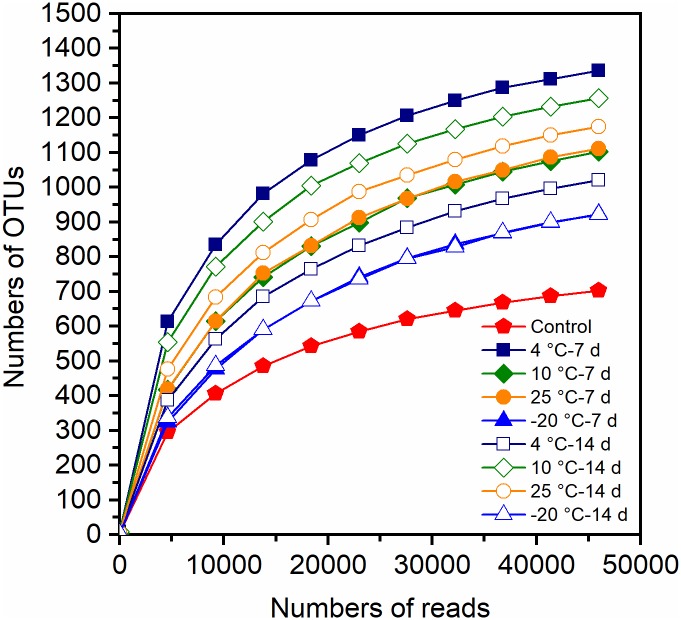
Rarefaction curve of Illumina HiSeq sequencing based 16S rRNA gene amplicon for the anode biofilm samples.

The observed species and coverage (>99.0%) of each of the MFCs were relatively stable when the sequences number reached 49892–55139, suggesting that the sequencing depths were sufficient to represent the presence of rare OTUs (Figure 4). The OTUs in different samples varied from 702 to 1336. The estimators of species richness, Chao1 and ACE indices, indicated that MFCs with pretreated inocula had a higher predicted species richness than did the control MFCs with untreated inocula (Table 1). Compared to the control MFCs, MFCs with pretreated inocula exhibited higher Shannon and Simpson values, except for the −20°C pretreated inocula (Table 1). It is obvious that MFCs with 4°C – 7-day- and 10°C – 14-day-pretreated inocula had the highest species richness, while the MFCs with −20°C pretreated inocula had the lowest richness. Thus, pretreatment in psychrophilic temperatures results in different species richness, and pretreatment at 4°C for 7 days and 10°C for 14 days can result in high richness of microbes. When the observed OTUs were similar between 7- and 14-day-pretreatments, the 14-day-pretreatment showed relatively higher diversity indices by enhancing evenness.

**Table 1:** Observed species and diversity indexes of the MFCs started up at 4°C with the inocula pretreated at different temperatures for 7 days and 14 days.

Samples	Reads	OTUs	Chao1	ACE	Shannon	Simpson
Control	55139	702	781.60	789.83	3.09	0.55
4°C – 7 days	54436	1336	1455.95	1453.27	6.11	0.90
10°C – 7 days	54366	1102	1236.29	1255.59	3.61	0.59
25°C – 7 days	51542	1111	1268.20	1277.80	3.65	0.60
−20°C – 7 days	52423	923	1055.12	1073.66	3.05	0.56
4°C – 14 days	52918	1021	1177.22	1183.06	4.79	0.87
10°C – 14 days	49892	1256	1380.49	1384.68	6.26	0.95
25°C – 14 days	53504	1174	1290.76	1308.80	4.60	0.74
−20°C – 14 days	53797	921	1056.96	1083.27	3.13	0.54

**FIGURE 5:**
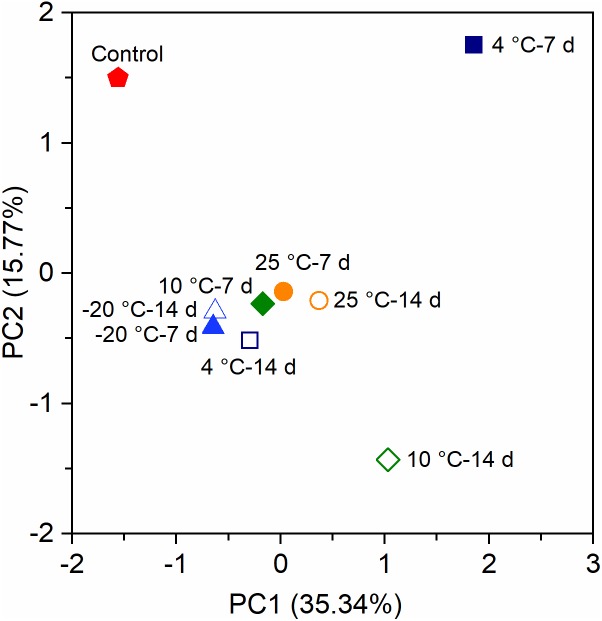
Principal components analysis (PCA) of the anode biofilms of MFCs at 4°C based on the operational taxonomic units (OTUs).

Principal components analysis (PCA) indicated that the bacterial communities of the anode biofilms in MFCs were categorized into four clusters based on OTUs (Figure 5). The control MFCs substantially separated from all MFCs with the pretreated inocula. Excluding the bacterial community of the control reactors, the community structures of 4°C – 7 days and 10°C – 14 days were vastly different from the others. There was obvious separation between different pre-acclimated periods at 4°C and 10°C, though not at −20°C and 25°C. The results implied that pretreated inocula at low temperatures (4°C and 10°C) have a substantial impact on the bacterial community structures.

### Temperature-Pretreated Inocula Shaped Bacterial Community Structure in MFCs

**FIGURE 6:**
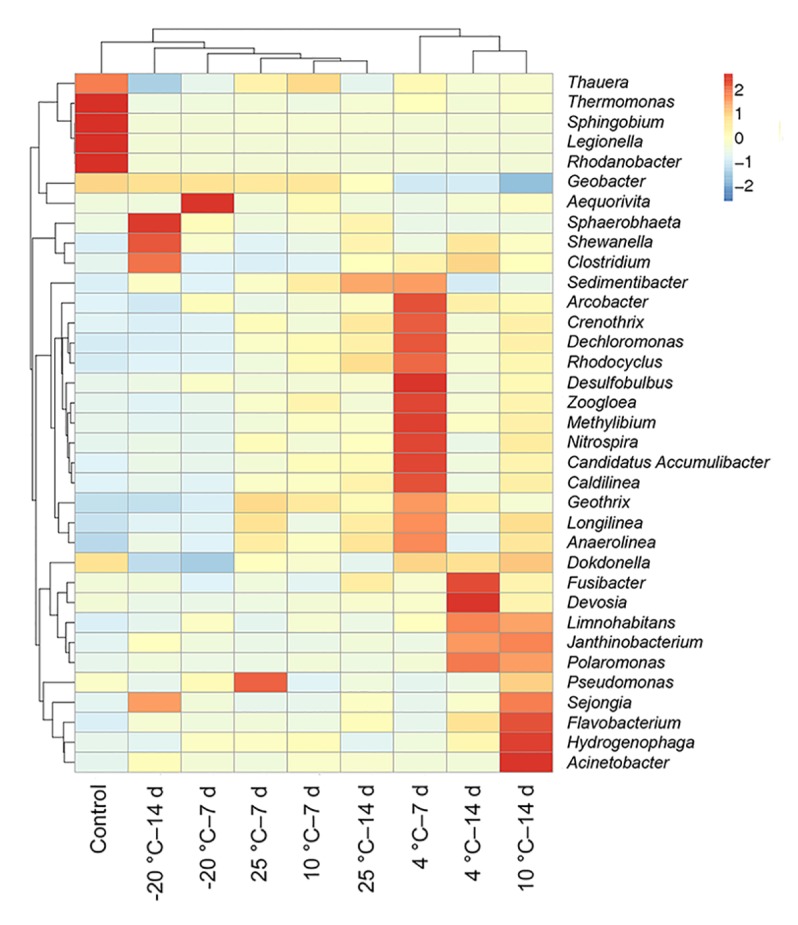
Hierarchical clustering and heatmap analysis of nine anode biofilms community structure were described at the genus level. The bar on the right represents the scale of the relative abundance.

The heatmap indicated that a difference in predominant populations between the control and the MFCs with pretreated inocula was present; the 4°C – 7-day-pretreated inocula differed from other pretreated MFCs (Figure 6). *Thauera*, *Thermomonas*, *Sphingobium*, *Legionella*, and *Rhodanobacter* were the differential populations in the control MFCs. *Desulfobulbus*, *Methylibium*, *Nitrospira*, *Candidatus Accumulibacter*, and *Caldilinea* were the most differential populations in MFCs with 4°C – 7-day-pretreated inocula, while differential populations in MFCs with 4°C – 14 days belonged to *Devosia* and *Fusibacter*.

**FIGURE 7:**
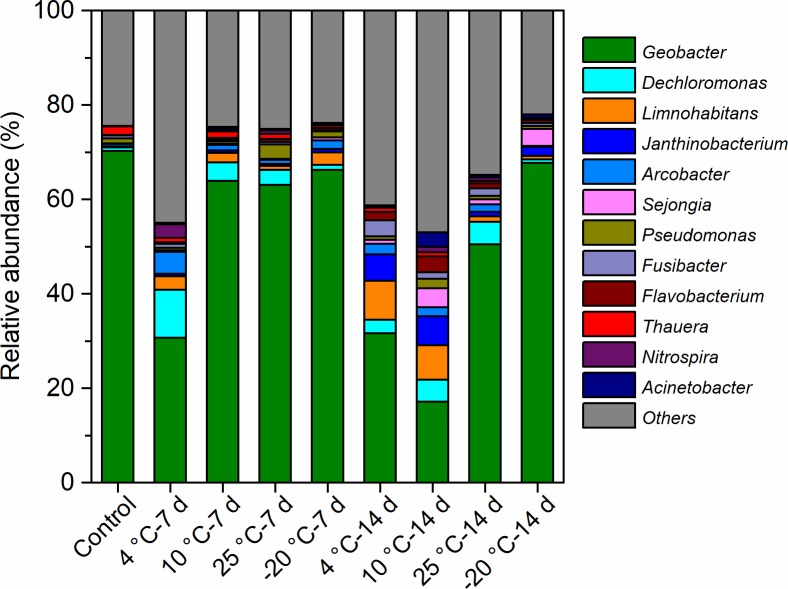
The predominant genera of the anode biofilms in the MFCs started up at 4°C with the inocula pretreated at different temperatures for 7 days and 14 days.

The most predominant genera of the MFCs were affiliated with *Geobacter*, *Dechloromonas*, *Limnohabitans*, *Janthinobacterium*, *Arcobacter*, and *Sejongia* (Figure 7). The relative abundance of *Geobacter* varied from 17 to 70% in the bacterial communities of the anode biofilms, which was high in MFCs with −20°C – 7-day- and −20°C – 14-day-pretreated inocula, indicating that *Geobacter* could be effectively enriched using −20°C pretreated inocula. On the contrary, the relative abundance of *Geobacter* was lower in MFCs with both 4°C – 7-day- and 4°C – 14-day-pretreated inocula compared with others, suggesting that a temperature of 4°C may allow more varied communities to grow. The statistical analyses indicated that *Geobacter* was significantly and positively correlated with Shannon and Simpson indices (Table 2). The relative abundance of *Dechloromonas* was higher in the 4°C – 7-day-pretreated inocula than in the others, and bacteria was positively connected with richness indices (observed species, Chao1, and ACE). *Limnohabitans* and *Janthinobacterium* were found in larger percentages in the 4°C – 14-day and 10°C – 14-day samples than other reactors, and the amount of *Limnohabitans* was positively correlated with the Simpson index. *Arcobacter* was found to be positively connected with the Shannon index (0.721^∗^), which had an amount that was also larger in 4°C – 7-day-pretreated inocula.

**Table 2:** Pearson correlations between the predominant genera and the diversity indexes.

Genera	Observed species	Chao1	ACE	Shannon	Simpson
*Geobacter*	−0.683	−0.69	−0.670	−0.954^∗∗^	−0.99^∗∗^
	0.058	0.069	0.069	0.000	0.000
*Dechloromonas*	0.919^∗∗^	0.910^∗∗^	0.907^∗∗^	0.783^∗^	0.659
	0.001	0.002	0.002	0.022	0.075
*Limnohabitans*	0.179	0.196	0.167	0.587	0.748^∗^
	0.687	0.641	0.693	0.126	0.033
*Janthinobacterium*	0.068	0.09	0.069	0.510	0.656
	0.873	0.832	0.872	0.196	0.077
*Arcobacter*	0.665	0.656	0.634	0.721^∗^	0.693
	0.072	0.078	0.091	0.044	0.057

*Two-tailed. ^∗^p < 0.05, ^∗∗^p < 0.01.*

## Discussion

### Current Generation by BES With Temperature-Pretreated Inocula

The preservation procedure of the inoculum has an important influence on the performance of psychrophilic MFCs. However, the procedure has been naturally neglected and very few studies have completely analyzed the mechanisms. In this study, the MFCs attained high COD removal rates of 73–91%, and the peak voltages all reached 0.48 V. The maximum power density attained by MFCs was 630–712 mW/m^2^ by inocula pretreated at 25°C. The power densities of the other MFCs in decreasing order were attained by inocula pretreated at 4°C, 10°C, and −20°C, with values of 526–650 mW/m^2^, 497–533 mW/m^2^, and 404–437 mW/m^2^, respectively. The power densities are higher than those found in a previous study that treated domestic wastewater under mesophilic-ambient temperature-phased MFCs (23–30°C), which obtained a maximum power density of 422 mW/m^2^ (Ahn and Logan, 2010). Another study found a dramatic increase of the highest power density from 425 mW/m^2^ at 0°C to 1,260 mW/m^2^ at 30°C, and found that the MFCs initially operated at 15°C hardly generated electricity, while the reactors that first started up at 30°C were effective in power generation when the temperatures decreased to 4°C or 10°C (Cheng et al., 2011). The coulombic efficiencies were higher than those in a study that examined the influences of low temperatures, indicating that the coulombic efficiency varied from 24 to 38% at 20°C and 4°C, with power densities ranging from 486 mW/m^2^ to 602 mW/m^2^. Moreover, there was a confusing phenomenon in which, even though the reactor conditions were the same, some MFCs could be easily started while others were unable to start. Our results indicated that this might be attributable to the fact that some inocula were used once they were collected, but other inocula were unintentionally kept in refrigerators for some days before inoculation. Although the MFCs were initially operated at 4°C, the power densities were even higher than in a previous study of MFCs (422 mW/m^2^) that treated domestic wastewater at 23–30°C (Ahn and Logan, 2010). On the contrary, it was also reported that MFCs can hardly generate electricity at a higher psychrophilic temperature of 15°C (Cheng et al., 2011). The reason for the contradiction in the results might be the influences of inocula preservation. Moreover, the present coulombic efficiencies (41–51%) were approximately two times higher than those found in a study that obtained coulombic efficiencies of 24–38% at 4°C, which might also be attributable to an effect of the inocula preservation.

### Inoculum Influences Start-Up of Psychrophilic BES

Performances of MFCs are mainly determined by electrode biofilms capable of EET (Logan et al., 2006; Malvankar et al., 2012). Direct start-up of BESs at temperatures below 5°C is difficult because of the slow growth of the electrode biofilm-composed psychrophilic exoelectrogens. The performance of MFCs decreased because of the decline of temperatures (Cheng et al., 2011). Various strategies have been used to enrich exoelectrogenic bacteria and overcome the problem of temperature (Lu et al., 2011, 2012b; Shrestha et al., 2019). Only one previous investigation reported for the first time the successful direct start-up and stable operations of MFCs and MFCs at 4°C via a sequential inoculation approach with a mixed-culture inoculum (Lu et al., 2011). Our study reports that the inoculum preservation approach can enhance the power output of MFCs at 4°C. The recent studies indicated that inocula from different sources influence the electricity generation of MFCs and the community structure of the anode biofilms at mesophilic conditions (Mei et al., 2015; Do et al., 2018). The current study explored the effects of the pretreated inocula of activated sludge from a wastewater treatment plant (WWTP) on the performance of MFCs at 4°C, but the inocula from other sources should be estimated in the psychrophilic BESs after pretreatment at different temperatures. The MFCs obtained higher power density after being preserved at 25°C, indicating that exoelectrogenic microbes pre-enriched at a mesophilic temperature successfully adapted to 4°C. The psychrotolerant bacteria isolated at 10°C was able to grow at temperatures as low as 4°C (Holmes et al., 2004). However, the electricity generation by a pure culture in BESs at 4°C has not yet been reported.

### Enrichment of Psychrophilic *Geobacter* at Low Temperature

The mesophilic *Geobacter* species has been widely investigated as a typical exoelectrogen (*Geobacter sulfurreducens* and *Geobacter metallireducens*) (Holmes et al., 2016; Tian et al., 2017). In this study, the enriched psychrophilic *Geobacter* species differed phylogenetically from mesophilic *Geobacter* species. The relative abundance of psychrophilic *Geobacter* in MFCs with inocula pretreated at 10°C and 25°C decreased with an increase of pretreatment period from 7 to 14 days, and the diversity and relative abundance of non-exoelectrogenic bacteria increased correspondingly. In contrast, there was not a large difference in the relative abundance of psychrophilic exoelectrogens at −20°C, because the growth and reproduction of exoelectrogens and non-exoelectrogens was presumably inhibited at −20°C. PCA analysis also revealed that the bacterial communities of MFCs with −20°C – 7-day- and −20°C – 14-day-pretreated inocula were similar. This suggests that the pretreatment period had a complicated impact on the enrichment of exoelectrogens and non-exoelectrogens at different pretreated temperatures. MFCs with 10°C – 14-day-pretreated inocula exhibited a lower relative abundance of *Geobacter* than MFCs with 10°C – 7-day-pretreated and untreated inocula, though they had a higher power density. The community structure and the relative abundance of *Geobacter* in MFCs with inocula pretreated at 20°C were similar, but the power densities were substantially different with different pretreatment periods. Therefore, the higher relative abundance of *Geobacter* is not the reason for a higher power density. Presumably, DNA from dead cells at 14 days preservation was amplified and sequenced, resulting in the overestimation of the relative abundance of *Geobacter*. These results imply that this phenomenon may be derived from the differences in species and the activity of EET of *Geobacter*. To estimate the EET activity of putative psychrophilic exoelectrogens, the electrode biofilms in MFCs with the inocula pretreated at different temperatures require further investigation using metatranscriptomic or metaproteomic approaches.

Moreover, because of short sequencing reads, it is worth mentioning that the next-generation sequencing technologies primarily determined OTUs at the genus-level. The real condition of the taxonomic level of species for *Geobacter* might be different depending on whether or not the bacterial proportions were the same in each sample. As far as we know, there are a number of different *Geobacter* species (Zhou et al., 2017), and the ability of EET is different. Single-molecule sequencing technology with long reads will provide a powerful tool in distinguishing *Geobacter* spp. at the species-level.

### Shaping Bacterial Community Structures by Temperature-Pretreated Inocula

Anaerobic psychrophilic microbes have only been discovered in natural environments (Lettinga et al., 2001). The discovery of groups of microbes that naturally prefer low temperatures or adapt quickly to psychrophilic temperatures is helpful for the advancement of biological technologies (Ahn and Logan, 2010). Mesophilic bacteria enriched in MFCs require longer periods of time to adapt in low temperatures, thereby causing the changes of community structures to be uncertain when the operation time is relatively short. Our study indicated that the response of exoelectrogens and non-exoelectrogens to temperature is substantially different, and so far only a putative psychrophilic exoelectrogen, *Geobacter* spp., has been identified in BESs (Lu et al., 2011, 2012a). A recent study has reported that psychrophilic *Geobacter* was enriched in MFCs at 7.5°C using arctic soil as the inoculum (Heidrich et al., 2018). PCA determined the bacterial communities of MFCs with inocula pretreated for 7 days and 14 days at 25°C were similar, but they were clearly separated at 4°C and 10°C for different pretreatment periods. Our study also provides another new approach for the fast start-up of psychrophilic BES at 4°C using preservation inocula beyond the sequential inoculation method.

## Conclusion

This study describes the effect of inocula pretreated at different temperatures on the electricity generation and microbiome in MFCs at low temperatures. Inocula pretreated at both psychrophilic (−20°C, 4°C, and 10°C) and mesophilic (25°C) temperatures were observed to enable fast start-up of MFCs at 4°C. After the start-up, the psychrophilic MFCs obtained the power densities of 404–712 mW/m^2^, the COD removals varied from 73 to 91%, and the coulombic efficiencies were 41–51%. Moreover, different pretreatment temperatures and periods both impacted the electricity generation and bacterial communities of the anode biofilms of MFCs. The pretreatment period had different effects on the enrichment of psychrophilic exoelectrogens and non-exoelectrogens in MFCs with inocula pretreated at different temperatures. In terms of power output of MFCs, the 7-day-pretreatments were generally more beneficial than were those of 14 days. In terms of temperature, all psychrophilic and mesophilic pretreatments achieved the similar peak voltage of 0.48 V. The majority of psychrophilic populations demonstrated the potential of the enrichment of *Geobacter* in MFCs with pretreated inocula. This study provides a simple method to start up psychrophilic MFCs, which have been understandably neglected by previous studies. The inocula pretreatment method can also be applied in the start-up of other psychrophilic BESs.

## Author Contributions

SL, BX, and DX designed the study and wrote the manuscript. SL, BX, BFL, BYL, and DX conducted the data analysis. All authors read and approved the final manuscript.

## Conflict of Interest Statement

The authors declare that the research was conducted in the absence of any commercial or financial relationships that could be construed as a potential conflict of interest.

## Footnotes

1 http://qiime.org

2 http://www.arb-silva.de
