# Supplementary material for: Neglected Effects of Inoculum Preservation on the Start-Up of Psychrophilic Bioelectrochemical Systems and Shaping Bacterial Communities at Low Temperature
Source: Front Microbiol. 2019 May 2;10:935. doi: 10.3389/fmicb.2019.00935 (PMC6507619; doi:10.3389/fmicb.2019.00935)
Supplement: Supplementary file 1 [file Data_Sheet_1.PDF]

Supplementary Materials

Neglected effects of inoculum preservation on the start-up of psychrophilic bioelectrochemical systems and shaping bacterial communities at low temperature

Sidan Lu [a, b, #], Binghan Xie [a, #], Bingfeng Liu [a], Baiyun Lu [a], Defeng Xing [a,*]

[a] State Key Laboratory of Urban Water Resource and Environment, School of Environment, Harbin Institute of Technology, Harbin 150090, China

[b] Department of Civil and Environmental Engineering, Louisiana State University, Baton Rouge, LA. 70803, USA

[*]Corresponding author. Tel: +86-451-86283123. E-mail address: dxing@hit.edu.cn

#Equal contribution.

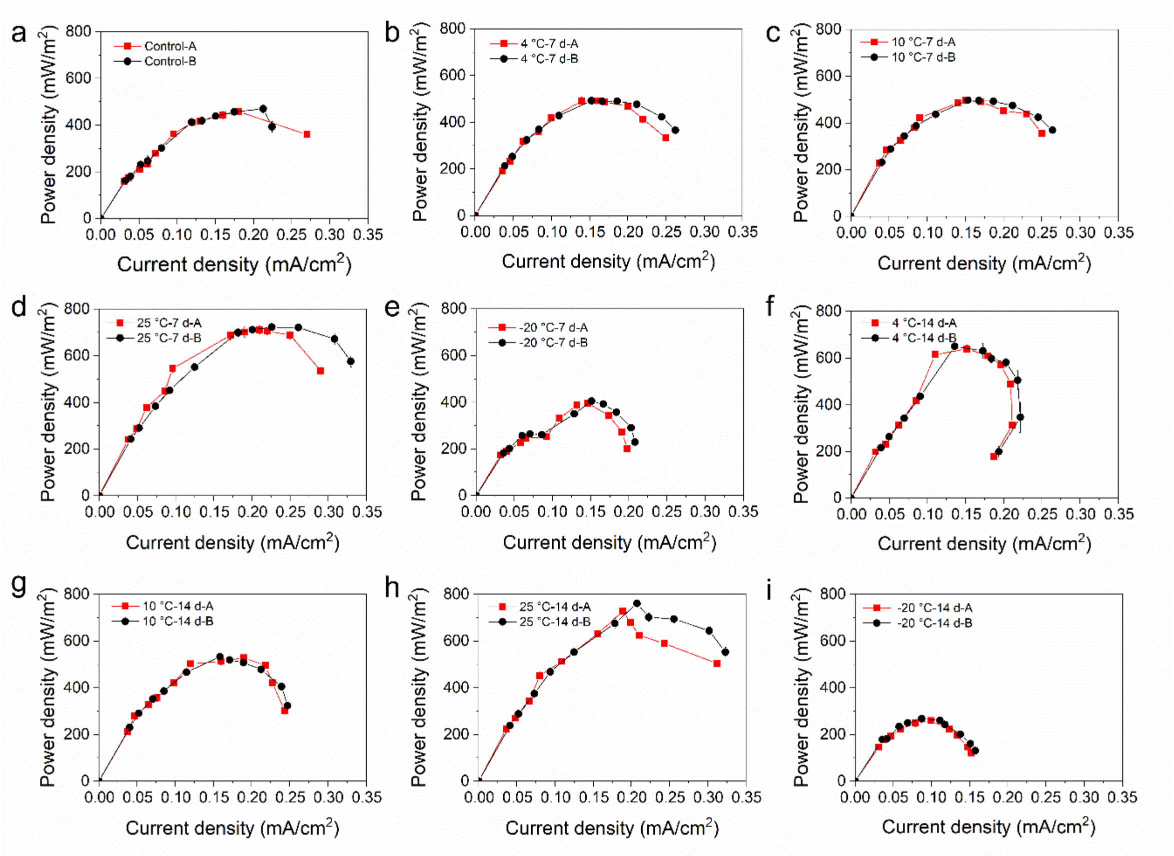

Fig. S1. Power density of duplicate MFCs which were started up at 4 °C with the inocula pretreated at different temperatures for 7 days and 14 days. Error bars represent standard deviation based on measurements from three batch cycles.
